# Cellulose Perversions

**DOI:** 10.3390/ma6041377

**Published:** 2013-03-28

**Authors:** João P. Canejo, Maria H. Godinho

**Affiliations:** CENIMAT/I3N, Materials Science Department, Faculty of Science and Technology, New University of Lisbon, Caparica 2829-516, Portugal; E-Mail: jpgc@campus.fct.unl.pt

**Keywords:** cellulose-based liquid crystals, electrospun helical fibers, biomimetic springs

## Abstract

Cellulose micro/nano-fibers can be produced by electrospinning from liquid crystalline solutions. Scanning electron microscopy (SEM), as well as atomic force microscopy (AFM) and polarizing optical microscopy (POM) measurements showed that cellulose-based electrospun fibers can curl and twist, due to the presence of an off-core line defect disclination, which was present when the fibers were prepared. This permits the mimicking of the shapes found in many systems in the living world, e.g., the tendrils of climbing plants, three to four orders of magnitude larger. In this work, we address the mechanism that is behind the spirals’ and helices’ appearance by recording the trajectories of the fibers toward diverse electrospinning targets. The intrinsic curvature of the system occurs via asymmetric contraction of an internal disclination line, which generates different shrinkages of the material along the fiber. The completely different instabilities observed for isotropic and anisotropic electrospun solutions at the exit of the needle seem to corroborate the hypothesis that the intrinsic curvature of the material is acquired during liquid crystalline sample processing inside the needle. The existence of perversions, which joins left and right helices, is also investigated by using suspended, as well as flat, targets. Possible routes of application inspired from the living world are addressed.

## 1. Introduction

Helical structures can be found in many natural systems, in scales that range from the nanometer to the macro-scale. These systems include hair, animal antlers or snail shells. The presence of helices can be a way to increase the superficial area or to enhance mechanical properties, among many other purposes. The existence of helices and coils in nature can be, in many cases, the consequence of differential growth, which results in the forced winding of the structure [1,2].

**Figure 1:**
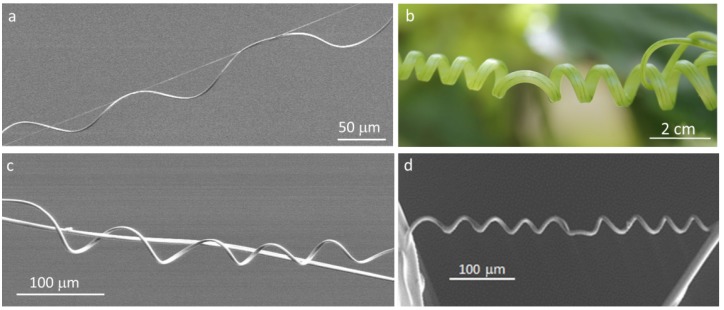
Helical shapes and perversions in biological systems: (**a**) fibers of spider webs (photo of spider silk produced by an unidentified spider); (**b**) cucumber tendril helices; (**c**) and (**d**) cellulose fibers electrospun from liquid crystalline solutions.

An interesting aspect about this winding phenomenon is the fact that natural helical structures can sometimes present not one, but two helices of opposite handedness, which are connected by a small straight linear segment (Figure 1). The area of the filament where the reversal of the helix handedness takes place is termed “perversion”. This type of helical shape separated by a perversion can be found in many natural systems and, perhaps, the tendrils of climbing plants represent one of the most common (Figure 1b). Darwin performed the first detailed study of plant tendrils, their perversions and motion [3]. He observed that twisted tendrils of climbing plants raised from seeds sent to him by a friend in 1858 moved in circumnavigation patterns over long periods of time in search of a support point [4]. By painting lines on the tendrils, Darwin recorded and visualized their motion and twist. He noticed that a tendril that did not find a point that could support the plant would form a spiral and that when a tendril attached itself to a support, would coil, forming a perversion [5].

The explanation of why perversions appear may be found in the fact that during the transition from a twisted rod to a helix, both ends of the fiber remain attached to fixed supports. Making a helix from a flexible rod that has a free end is simple; it is enough to rotate the end that is free to move. When none of the extremities are able to rotate, the construction of a helical shape is a much more challenging task. A quantitative approach to the problem was performed by Goriely* et al.* for the twisting of plant tendrils after they reach a support [6,7,8].

When tendrils find a support point, they became hard and strong as they grow. The twist of the tendril gives the necessary elasticity for the plant to survive heavy loads, like the ones caused, for example, by strong winds. However, since the two extremities are fixed and cannot rotate, the total torsion of the tendril must remain constant, and the twisting causes the formation of two helices, where the handedness of one helix cancels the opposite handedness of the other, keeping the total twist constant. The model proposed by Goriely* et al.* describes the hand reversal of helical twist for elastic and slender filaments. While the filament is under tension, it is twisted in a linear configuration. If the applied tension is released, eventually the linear shape becomes unstable, and the value of the tension at which the transition from a straight line to a helical structure happens is known as the critical tension. The release of the elastic energy of the filament leads to a change from the linear shape to the formation of two helices, of reverse handedness, by rotation of the perversion. The number of turns in the helices is a function of the applied tension as the rotation of the perversion results in an increased number of turns in the helices. The value of the critical tension is given by ∅c=K/√Γ, where K represents the intrinsic curvature of the filament and Γ = 1/(1 + σ) measures the relation between the bending and rotation coefficients, with σ representing the Poisson coefficient. In the case of plant tendrils, the differential growth of diverse areas in the tendril is responsible for the intrinsic curvature of the system. Recently, it was reported that the intrinsic curvature of some old plant tendrils species can occur via asymmetric contraction of an internal fiber ribbon of specialized cells, which generates different shrinkages of the inside relative to the outside tendril ribbon, allowing its overwinding, rather than unwinding, under tension [9].

Cellulose, the main constituent of plant cell walls, seems an ideal material to mimic the micro- and nano-structures that are present in many natural living systems [10,11]. Some years ago, it was found that concentrated solutions of cellulose derivatives displayed colors that changed with concentration and viewing angle, which was attributed to a chiral nematic structure [12]. The anisotropic nature of cellulose solutions gives them unique characteristics that have impact, for example, in the way the viscosity changes with polymer concentration. For low polymer concentrations, while in the isotropic phase, the viscosity of the solutions increases with the increase of the polymer fraction. However, for higher polymer concentrations, corresponding to the formation of the liquid crystalline phase, the increase in concentration results in a decrease of the viscosity. This was attributed to the spontaneous orientation of the mesogenic cellulose segments above the critical concentration [13]. The viscosity drop, associated to the liquid crystalline phase appearance, is an important effect that one can take profit from when working with relatively high concentrated cellulose based solutions. Solid chiral nematic films showing iridescent colors, as well as sheared films, were also prepared from hydroxypropyl cellulose (HPC) by quenching from the melt [14,15], and electrospinning was also used to produce continuous helical fibers, with diameters ranging from few nanometers to hundreds of microns, generating mats with a very high surface area [16,17]. The electrospinning setup can have many different configurations and components, but the basic setup only needs three major components: a high-voltage power supply, a metallic needle or a capillary tube and a grounded collector or target [18]. The solution from which the fibers will be produced is placed inside a syringe attached to a metallic needle; a syringe pump is used to force the solution through the metallic needle at a controlled and constant flow rate. A high-voltage power supply that is connected to the tip of the needle and to the target is used to apply a voltage between these two components that, usually, is in the range of a few kV to 30 kV.

During the fiber production process, the solution flow rate is set with the aim of always having a droplet on the tip of the needle. This droplet assumes a spherical shape in order to minimize its superficial tension. When the electric field is applied between the metallic needle and the collector, the formation of electrostatic charges on the surface of the droplet is induced; these increase as the intensity of the applied field increases. The droplet is, therefore, under the action of two electrostatic forces; one is repulsive, due to the superficial charges, and the other is exerted by the electric field. As the electrical field increases, the droplet becomes unstable and is deformed through the force generated by the superficial charges, forming a conical shape known as Taylor’s cone [19]. Above a critical threshold, the electrostatic repulsion forces become stronger than the superficial tension of the polymeric solution. Eventually, a jet of electrically charged solution breaks free and heads towards the target. During the travel from the needle to the collector, the polymeric jet, subjected to a very high shear rate, is stretched, until it forms a fiber with a final diameter several times smaller than the inner diameter of the metallic needle [20]. Initially, the path is linear, but, because of instabilities, due to electrostatic interactions between the electric field and the superficial charges of the jet, the trajectory becomes random before it reaches the target. This makes fiber production unstable and problematic to deposit on specific areas of the target. As the electrospun fibers move towards the target, a sufficient amount of solvent evaporates, ensuring that the fibers reach the collector with the necessary rigidity to support their own height, maintaining the shape. One of the characteristic of electrospinning is the fact that the diameters of electrospun fibers are distributed along a range of diameters instead of having one fixed diameter value.

The thickness of membranes obtained using electrospinning is a function of the deposition time; this is a continuous process, and the produced fibers are deposited over previously electrospun fibers.

Obtaining micro- and nano-helical springs from electrospun fibers was also reported, either by taking advantage of the electrical field during fiber production [21] or by the use of a two-component solution [22].

In order to better understand the impact of the intrinsic curvature found in cellulose-based electrospun filaments (Figure 1b), which mimic the structures found at the micro-scale in spider webs (Figure 1a) and in tendrils three to four orders of magnitude larger (Figure 1b), we followed their trajectories when produced from isotropic and anisotropic solutions. The behavior of cellulose microfibers, with two opposite-handed helices connected by a helical perversion, when clamped at both ends and pulled axially, upon electron-beam exposure, was recorded and analyzed, taking into account the formation of a rigid core disclination forming off-axis along the filament.

## 2. Results and Discussion

**Figure 2:**
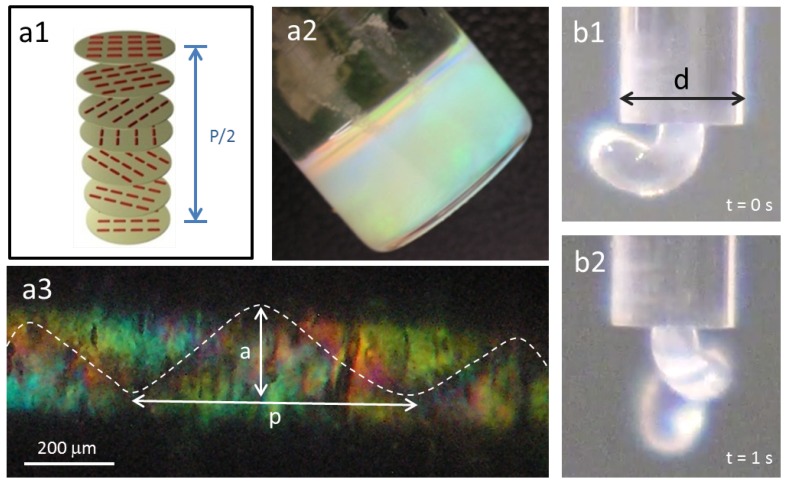
Cholesteric liquid crystalline phases in cellulosic solutions: (**a1**) schematic of the cholesteric structure; (**a2**) cholesteric hydroxypropyl cellulose (HPC) solution; (**b1**) and (**b2**) show HPC a2 solution when exiting a capillary tube, d = 1.7 mm; (**a3**) polarizing optical microscopy (POM) picture, between cross polarizers, of the a2 solution after shear inside the capillary, ($\dot{\gamma }$ = 18 s^−1^) (a ~ 188 μm and pitch p ~ 625 μm).

In our work, the material used to prepare the helical fibers is a very well-known cellulose derivative that does not dissolve in water, but generates a liquid crystalline phase in dimethyl acetamide. The anisotropic solution showed an iridescent color, and the maximum peak wavelength (λ_0_) reflected by the sample for incident light normal to the surface may be expressed as, λ_0_ = n_e_ P cos*θ* [15], where n_e_ is the refractive index equal to 1.33 for this system, P = 329 nm, the helical pitch, and *θ*, the angle between the light propagation direction and the helix axis (*θ* = 0°) (Figure 2a,b). The anisotropic solution, 60% (w/w) polymer content and an isotropic solution, 10% (w/w) polymer content, were sheared (shear rate equal to 18 s^−1^) in a glass capillary (diameter equal to 1.7 mm), and the flow inside the capillary and the jet at the end of the tube was recorded. For liquid crystal solutions, as observed previously [23], the jet showed spontaneous curvature and torsion (Figure 2b), while a straight one was obtained for isotropic solutions. POM images taken along the glass capillary, between cross polarizers, revealed a helix linear topological defect (disclination) (Figure 2c). In literature, similar observations were described for this cellulose derivative solution, and the data obtained was supported by MRI measurements, which allowed the association of the line defect to a local mechanical stiffness of the material [23].

**Figure 3:**
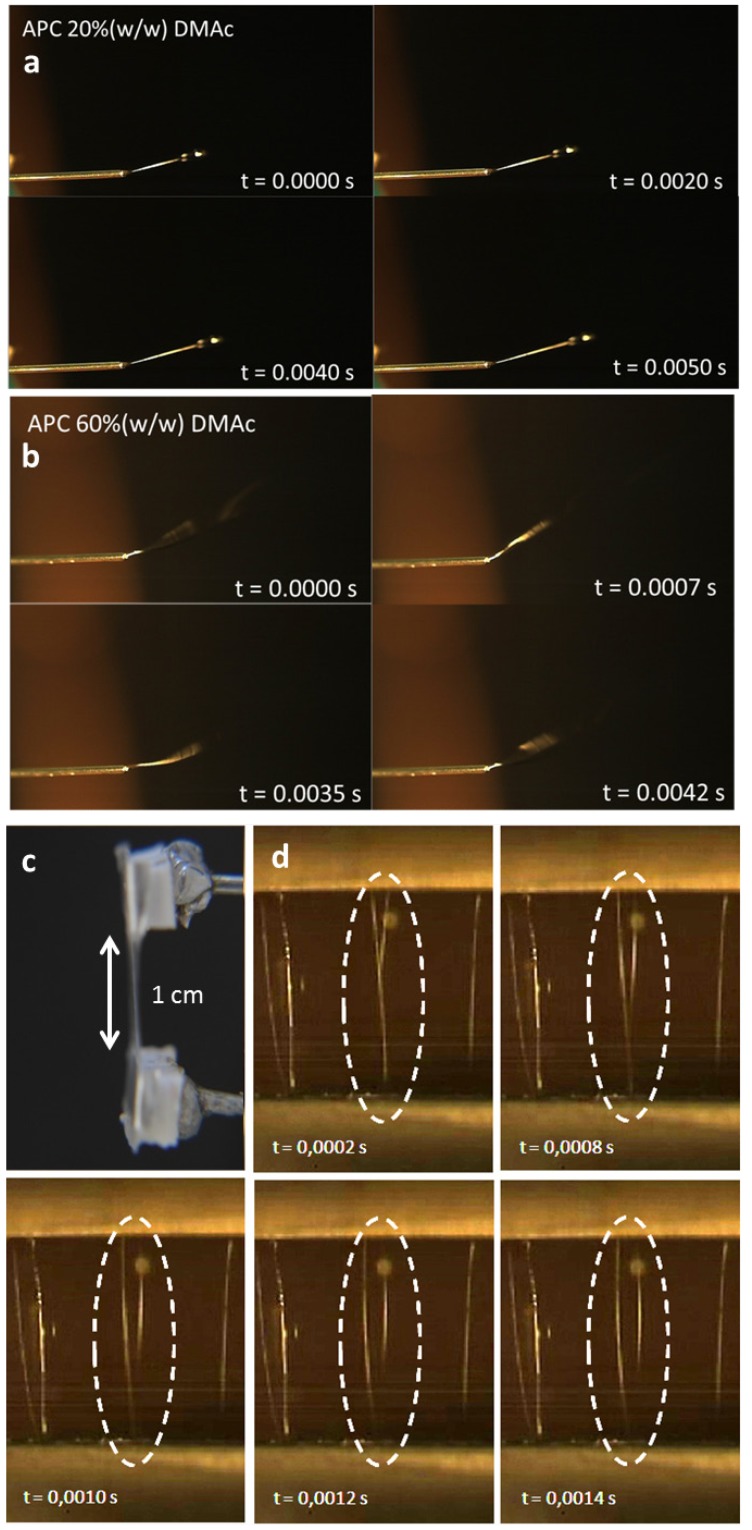

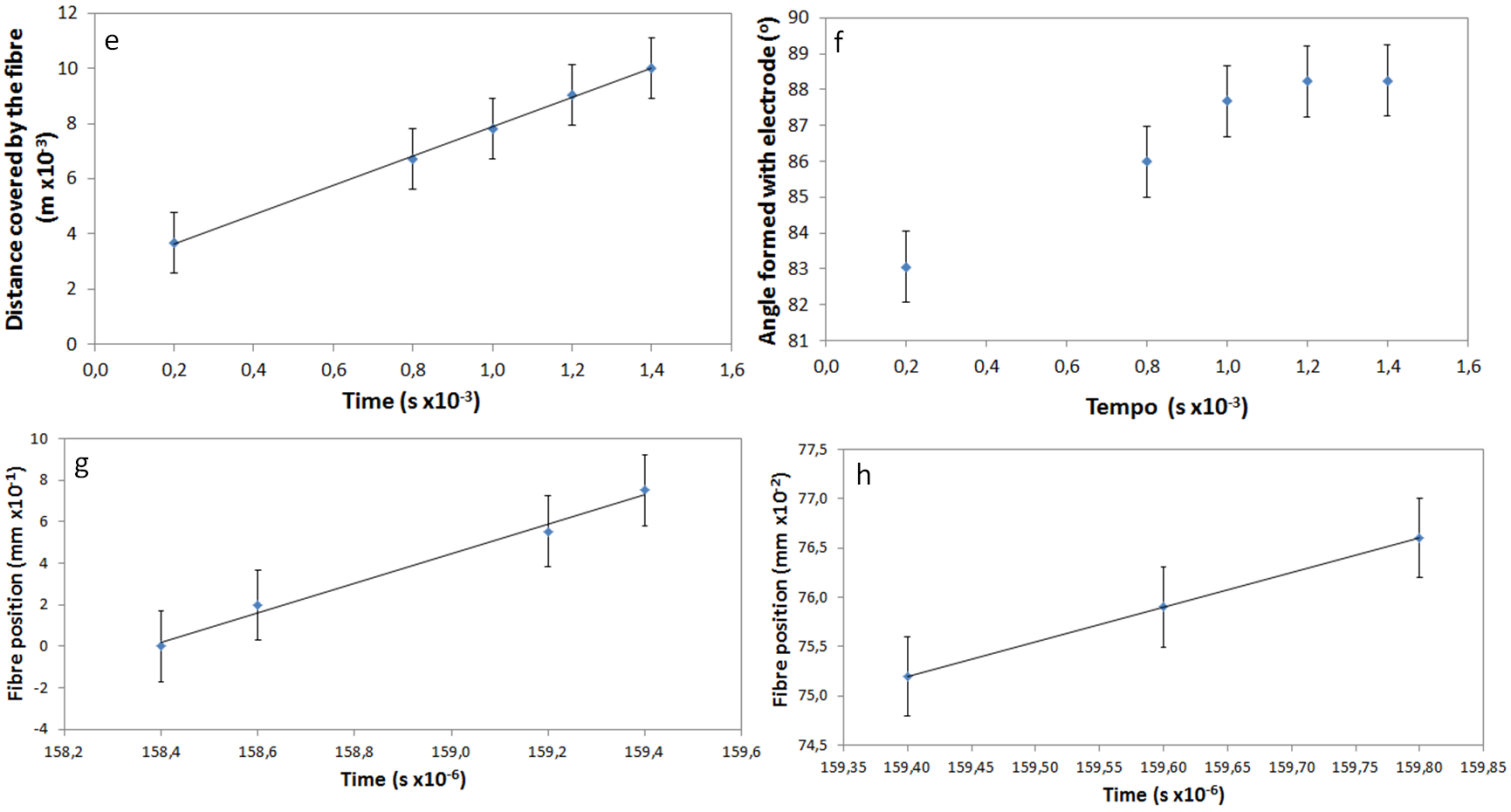
Frames were taken form a movie of the tip’s needle during electrospinning of an isotropic (**a**) (Figure S1) and a liquid crystal (**b**) (Figure S2) solution; (**c**) split electrode target, a gap of 1 cm and fiber deposition (**d**) and characteristics (**e**–**h**) as a function of time.

In order to follow the mechanism of the fiber formation, its trajectories during electrospinning, as it travels from the needle towards the target, were recorded using a high-speed camera. The isotropic and anisotropic solutions behavior was considered. The results show that fibers, produced from the isotropic cellulosic solution, leave the tip of the needle in a linear trajectory (Figure S1) (Figure 3a)) that shows the characteristic instabilities associated with the electrospinning technique only after traveling a distance of several centimeters [24,25]. In contrast, fibers produced from the liquid crystalline solution leave the needle tip in a circular trajectory (Figure S2) (Figure 3b) that can be attributed to the intrinsic curvature of the system acquired by the use of a high shear rate associated to the liquid crystalline characteristics of the material [26]. These circular movements seem to mimic the first stage of the tendrils development; as they grow, the tendrils circumnavigate. That is, the tip of the tendril moves in large loops in space by completely rotating on itself until it touches a support, such as a trellis, a pole or a branch. If the circumnutation does not result in a contact, the tendril eventually dries out and falls off the stem [27].

The high-speed camera was also used to record the trajectories of the electrospun cellulosic fibers during deposition on a split target consisting of two-parallel metallic bars. The analysis of the recorded movie revealed that during collection, the fiber ends do not reach the two electrodes simultaneously. Instead, the fibers are attached initially by one of its ends at one of the metallic bars, while the other end moves in the direction of the second metallic bar (Figure 3b). This behavior was already reported for fibers electrospun from a polyethylene oxide solution [28]. The obtained fibers were deposited parallel to one another, perpendicular to the electrodes and suspended between the two electrodes. The influence of the split electrode in the orientation of the fibers was studied [29], and it was found that the formation of electrostatic forces is responsible for stretching the fibers before the deposition. When the electrically charged fiber enters the vicinity of the electrodes, it induces the formation of electrical charges of opposite sign on the surfaces of the electrodes; the portions of the fiber closest to the electrode are pulled with stronger force. The strength of this electrostatic force, which is due to Coulomb interactions, is proportional to the square of the distance between the electrical charges and is stronger in the extremities of the fibers—the areas that are closest to the electrodes. Since this force has opposite directions on each of the electrodes, the result is the stretching of the fibers and their deposition in a straight line.

Using frames taken from the movie, it was possible to determine the time elapsed as the free end of the fibers passes over the gap between the two electrodes. This is a very fast movement, where the fiber connects the electrodes, which are separated by 1 cm, in less than 1 millisecond. The relation between the position of the fiber and the corresponding time is represented in the plot of Figure 3c. The linear variation of the distance travelled by the fiber with time means that the speed of the fiber is constant, and its value was determined as 5.3 m/s.

Electrostatic repulsion, between the fiber that is being deposited and the fibers that are already connecting the two electrodes, can be observed as the free end of the fiber moves toward the second electrode. Unlike what occurs with flat targets that promote a rapid discharge of the electrospun fibers, when deposited on split electrodes, the fibers maintain the electrostatic charge for longer times [29]. The speed at which the previously deposited fibers are moved from their original position, due to electrostatic repulsion, was calculated, and it was found to have two different mechanisms. Initially, the fibers change position with a constant speed of 0.709 m/s that lowers to 0.035 m/s near the end of the movement (Figure 3e,f).

The alignment of the fibers during the electrospinning process, in relation to the electrodes, was also determined from the recorded movie by measurement of the angle formed by the fiber and one of the electrodes. These values as a function of time are represented in Figure 3d, considering that a fiber parallel to the electrodes forms an angle of 0° and a fiber perpendicular with the electrodes corresponds to an angle of 90°. It is possible to observe that the speed of alignment decreases when the fiber is near its final position. Perfect alignment of the fibers was never observed; nevertheless, good alignment was achieved, with the values of the angles formed between fiber and electrode as high as 88.2°.

In the case of split electrodes, the final position of the deposited fibers is the result of the attraction force that stretches the fibers and the repulsion force that tends to align them [30]. For small electrode gaps (<1 mm), the attractive forces dominate, but became less important as the gap increases. For bigger distances between electrodes, the electric field is weaker in the center of the gap, and the repulsion forces are responsible for the orientation of the fibers [31]. Smaller gap distances result in thinner fibers. Considering that the electric field is distorted by the presence of split electrodes [32], it is possible to demonstrate that the minimal energy corresponds to fibers aligned parallel to each other. The degree of alignment was found to decrease with time; fibers deposited after 1 h of electrospinning are less aligned than fibers deposited after a few minutes. This could be the consequence of the total or partial discharging of previously collected fibers that no longer make any contribution for the orientation of the incoming fibers.

These results indicate that the fibers, during the production process, are deposited under tension. If this tension is released, which can be accomplished by approaching the electrodes, the fibers curl spontaneously, forming a helical structure.

**Figure 4:**
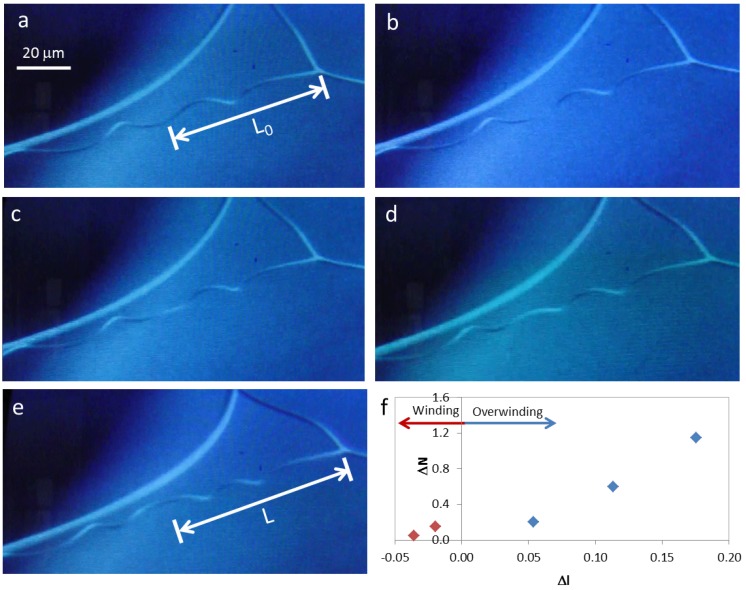
Winding and overwinding of a cellulose microfiber during SEM observation (**a**) to (**e**); (**f**) change in the number of turns present in each helix, ΔN, with scaled displacement, Δl.

SEM observations of electrospun fibers, after removal of the applied tension, revealed the formation of helical structures and perversions (Figure 1c,d and Figure 4). The helical twist involving the creation of a perversion may help to explain why the observed helices formed by the electrospun anisotropic cellulosic fibers do not rotate all with the same handedness. Since the cellulose-based solution from which the anisotropic fibers were electrospun has a cholesteric structure, whose helices have a right-handed twist [33], the expected result was that all the fibers should have the same helical right-handed twist. The shapes observed are curiously similar to the ones found in fibers taken from a spider web, also in the micro-scale, and to the ones present in plant tendrils in the macro-scale. This means that the systems described show intrinsic curvature, and the formation of helices and perversions should share the same physical model, even if they belong to different length scales.

The fibers that make a spider web represent one of the most interesting materials that can be found in nature. These fibers are biodegradable, very light and have excellent mechanical properties [34], which are achieved by means of environmentally friendly processes that use water as a solvent and occur at room temperature [35]. Like the cellulose liquid crystalline microfibers electrospun in this work, also spider web fibers are produced from a lyotropic liquid crystalline phase [36]. They consist of protein chains, which contain complex sequences of different amino acids crystallized into β-sheet crystals, with a z-shaped conformation [37]. Production from the liquid crystal phase seems to allow not only the orientation of the chains that is responsible for the mechanical characteristics needed for the web to catch flying prey, but also fiber production involving very low tensions.

During SEM observation of a suspended cellulosic fiber, it was possible to see rotation of the perversion, due to electronic beam exposure (Figure 4a–e), which led to an increase in the number of turns in the fiber. Measurement of the distance from the middle of the perversion to one of the fiber extremities gives an indication of how the length of the fiber changes during observation. Results show that a sequence of two different mechanisms could be in action. Initially, the rotation of the perversion is associated with a decrease in the fiber length (Figure 4f). This is due to the releasing of elastic energy that promotes the winding of the fiber as the tension decreases, which is in agreement with Goriely’s model. However, for longer times, the rotation of the perversion happens simultaneously with an increase in the fiber length (Figure 4f) and the fiber overwinds. This effect could have a relation with a softening of the polymer matrix caused by the heating that is induced by the electronic beam compared with the stiffness of the disclination line defect. The mechanism observed for longer times is similar to that recently described for both old *Cucumis sativus* (cucumber) and *Echinocystis lobata* (wild cucumber) tendrils [9].

The non-woven micro/nano-mats, formed by helices, which can overwind instead of unwind upon tension, open new perspectives in the production of high surface materials that can increase their performance under working conditions.

## 3. Experimental Section

### 3.1. Preparation of the Solutions

Hydroxypropyl cellulose (HPC) was purchased from Sigma-Aldrich ($\bar{M}$w = 100.000; ($\bar{M}$_S_) = 3.5) and was used as received. HPC solution in distilled water with a concentration of 63% (w/w) was prepared at room temperature. Acetoxypropyl cellulose (APC) was synthetized according to the procedure described in [38]. An anisotropic cholesteric solution in anhydrous dimethylacetamide (DMAc), purchased from Aldrich, with a concentration of 60% (w/w) was prepared by weighing the APC and dissolving it completely in the required amount of DMAc in order to obtain the chosen concentration. The solution was allowed to sit for one week, was stirred and was allowed to sit for another week before being used.

### 3.2. Electrospinning of APC Solution

The APC solution was transferred into a 1 mL syringe fitted with a 23-gauge metallic needle, which was placed on an infusion pump (KDS100) to ensure a constant feed rate of 0.04 mL/h. The metallic needle was electrically connected to a conducting ring, positioned coaxial with the needle tip in order to obtain a more uniform electric field, and both components were connected to the high-voltage power supply (Glassman EL 30kV) positive out-put. In order to obtain suspended and aligned fibers, the collector used has two split electrodes; two metallic strips of aluminum parallel to each other and separated by a 10 mm gap. The electrodes were mounted on a V-shaped support that is flexible enough to accommodate small deformations, so that the distance between electrodes can be reduced in order to remove the tension in the electrospun fibers. The target was grounded by connecting the aluminum to the high-voltage power supply. After the electric field was generated, the anisotropic solution was accelerated towards the collector placed 15 cm from the needle. The applied voltage was 15 kV. After the collection of the fibers, the target was kept under vacuum overnight to ensure that the solvent was fully evaporated.

### 3.3. Morphological Characterization

Samples of electrospun fibers and spider web silk were observed by means of Scanning Electron Microscopy (SEM) after being sputter coated with gold. Observations were made using a SEM DSM962 from Zeiss with an acceleration voltage of 5 kV. Fiber dimensions were measured with ImageJ software, version 1.44p. Optical photographs were taken with a Casio Exilim EX-F1digital camera.

### 3.4. Extrusion of APC Solution

APC solution was poured into a glass capillary with an inner diameter of 600 μm. By use of the infusion pump, the solution was forced through the tube at a flow rate of 4 mL/h, higher than the critical shear rate necessary to obtain a jet that shows intrinsic curvature [23]. In order to achieve gravitational pull, the setup was mounted vertically. A digital camera was used to record the formation of jets as solution exits the capillary tube.

### 3.5. Measurements of the Selective Reflection Peaks

The wavelengths (λ_0_) of the maximum selective reflection peaks were recorded with a Jobin Yvon monochromator H10 Vis mounted on the microscope stage, equipped with a photomultiplier and a chart recorder. Six measures were obtained for each sample. The film’s textures were observed using a POM Olympus BH2 in transmission and reflection mode coupled to a Canon EOS 550D digital camera.

### 3.6. Spider Web Collection

The spider web used in this work for SEM observation was collected in the University campus during the spring season, while suspend in the same place where it was woven by an unidentified spider.

## 4. Conclusions

Cellulose-based liquid crystalline solutions can generate jets and fibers with intrinsic curvature, which arise from stiff line defects (disclinations). Due to the intrinsic curvature of the system micro-jet swinging, as well as micro/nano-fiber winding and overwinding, self-motions can be observed and controlled by adjusting the temperature and electrospun experimental parameters. It seems that these cellulose-based materials not only mimic at the micro/nano-scale the helical shapes observed, for example, in tendrils, but also their motion and mechanical adaptability can be reproduced. The same mechanism is in action for all these systems, providing that the same physical model, proposed for elastic filaments with intrinsic curvature, could be applicable.

The smart cellulose-based non-woven mats produced, which respond to external stimuli, are expected to play a significant role in a new generation of nano- and micro-devices, because of their capacities as separation media, actuators and host materials for drug delivery.

## Supplementary Materials

Supplementary File 1
