# Supplementary material for: Cellulose Perversions
Source: Materials (Basel). 2013 Mar 28;6(4):1377–90. doi: 10.3390/ma6041377 (PMC5452322; doi:10.3390/ma6041377)
Supplement: Supplementary File 1 [file materials-06-01377-s001.pptx]

## Slide 1
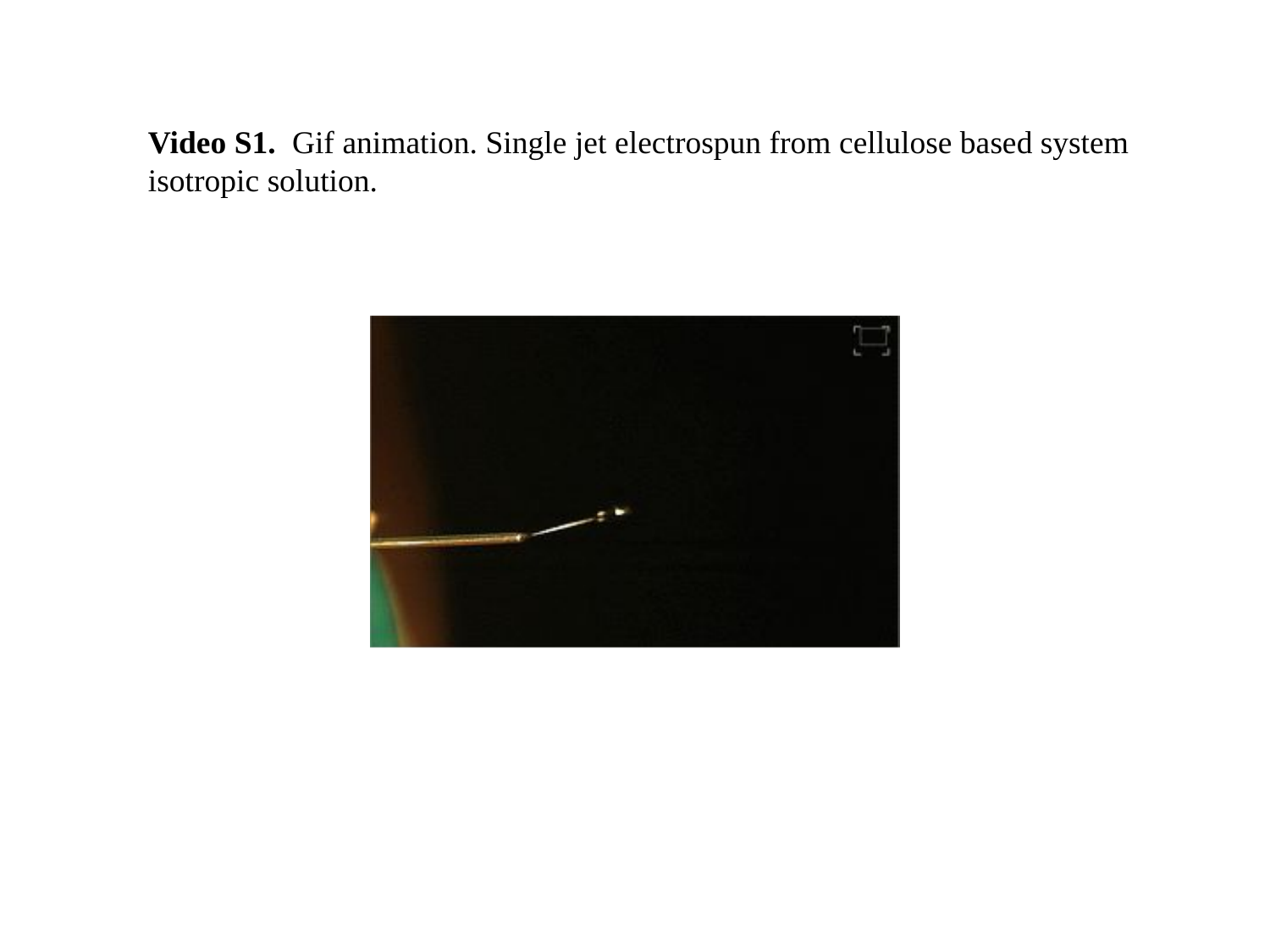

Video S1. Gif animation. Single jet electrospun from cellulose based system
isotropic solution.

## Slide 2
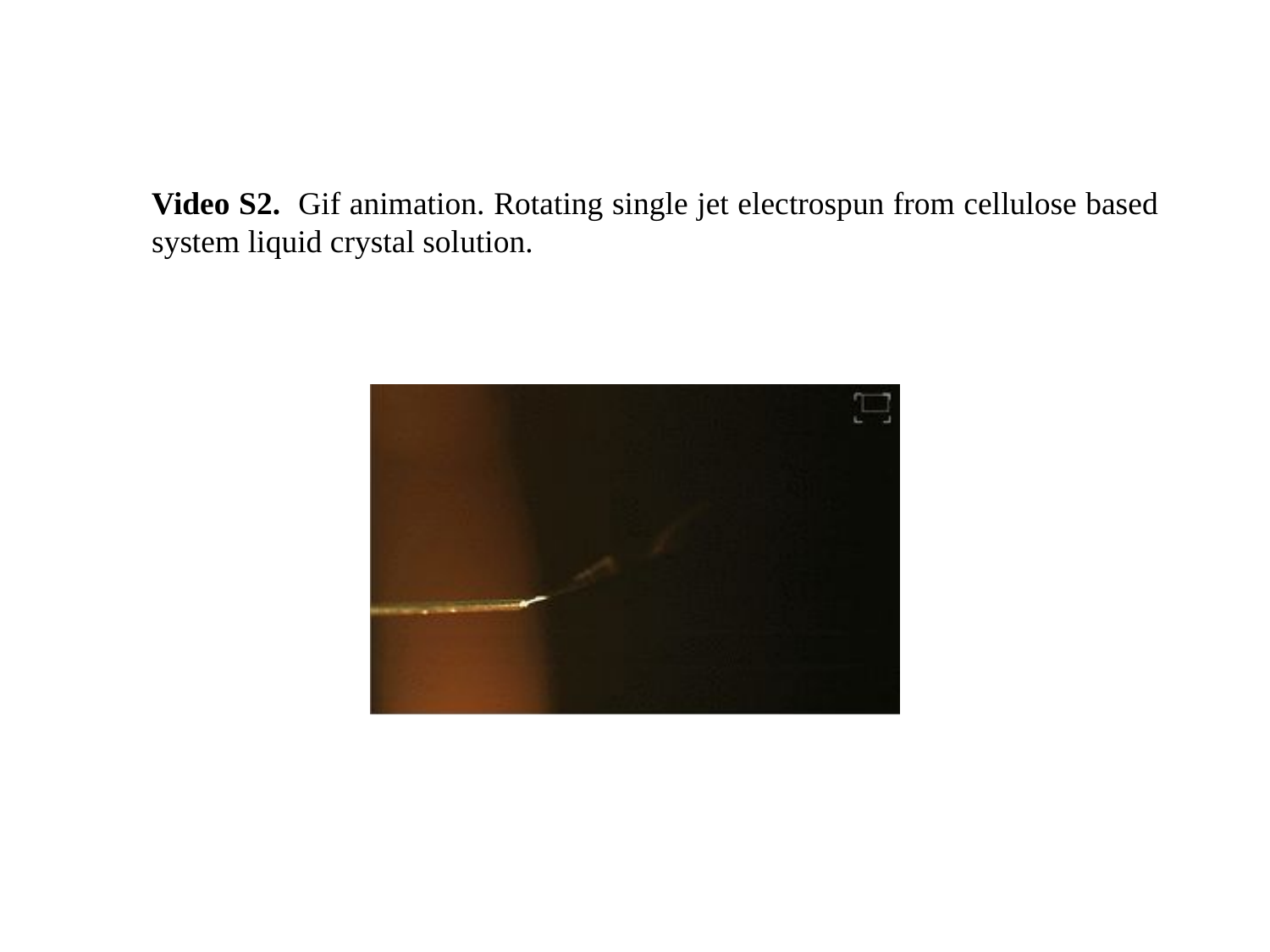

Video S2. Gif animation. Rotating single jet electrospun from cellulose based system liquid crystal solution.
